# Habitat transitions alter the adaptive landscape and shape phenotypic evolution in needlefishes (Belonidae)

**DOI:** 10.1002/ece3.6172

**Published:** 2020-03-06

**Authors:** Matthew A. Kolmann, Michael D. Burns, Justin Y. K. Ng, Nathan R. Lovejoy, Devin D. Bloom

**Affiliations:** ^1^ Department of Biological Sciences George Washington University Washington DC USA; ^2^ Friday Harbor Laboratories University of Washington Friday Harbor WA USA; ^3^ Cornell Lab of Ornithology Cornell University Museum of Vertebrates Ithaca NY USA; ^4^ Department of Biological Sciences Western Michigan University Kalamazoo MI USA; ^5^ School of Aquatic and Fishery Sciences University of Washington Seattle WA USA; ^6^ Department of Biological Science University of Toronto Scarborough Toronto ON Canada; ^7^ Department of Biological Sciences & Institute of the Environment and Sustainability Western Michigan University Kalamazoo MI USA

**Keywords:** Beloniformes, body size, elongation, marine–freshwater transitions, miniaturization

## Abstract

Habitat occupancy can have a profound influence on macroevolutionary dynamics, and a switch in major habitat type may alter the evolutionary trajectory of a lineage. In this study, we investigate how evolutionary transitions between marine and freshwater habitats affect macroevolutionary adaptive landscapes, using needlefishes (Belonidae) as a model system. We examined the evolution of body shape and size in marine and freshwater needlefishes and tested for phenotypic change in response to transitions between habitats. Using micro‐computed tomographic (µCT) scanning and geometric morphometrics, we quantified body shape, size, and vertebral counts of 31 belonid species. We then examined the pattern and tempo of body shape and size evolution using phylogenetic comparative methods. Our results show that transitions from marine to freshwater habitats have altered the adaptive landscape for needlefishes and expanded morphospace relative to marine taxa. We provide further evidence that freshwater taxa attain reduced sizes either through dwarfism (as inferred from axial skeletal reduction) or through developmental truncation (as inferred from axial skeletal loss). We propose that transitions to freshwater habitats produce morphological novelty in response to novel prey resources and changes in locomotor demands. We find that repeated invasions of different habitats have prompted predictable changes in morphology.

## Graphical abstract

Can we predict whether fishes will get larger or smaller body shape and size in saltwater or freshwater? We found that when needlefishes (Belonidae) evolve in freshwater habitats, they always get smaller in size. These changes are predictable, and we show that the manner of selection for body size and shape is fundamentally different among marine and freshwater habitats.

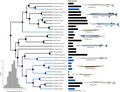

## 1 INTRODUCTION

Understanding drivers of uneven diversity among clades is a fundamental goal of evolutionary biology. While many studies focus on species diversity patterns (Benton, 2001; Grosberg, Vermeij, & Wainwright, 2012; Sahney, Benton, & Ferry, 2010; Wiens, 2015), there is also an exceptional disparity in phenotypic diversity among clades. Recent studies have demonstrated shifts in habitats can influence the rate and mode of morphological diversification (Collar, Schulte, O’meara, & Losos, 2010; Price, Holzman, Near, & Wainwright, 2011; Price, Tavera, Near, & Wainwright, 2013). Major transitions including the shift from water to land by early tetrapods and the advent of powered flight in pterosaurs, birds, and bats profoundly influenced the evolutionary trajectory of these clades (Balanoff, Smaers, & Turner, 2016; Benson, Butler, Carrano, & O'Connor, 2012; Kawano & Blob, 2013; Standen, Du, & Larsson, 2014). The effect of habitat shifts on clade dynamics is likely amplified when there is an associated change in habitat complexity (Benton, 2001), with more complex habitats likely driving greater phenotypic diversity (Price et al., 2013).

Within the aquatic realm, one of the most fundamental ecological divisions is between marine and freshwater habitats (Lee & Bell, 1999). While numerous lineages have crossed the marine–freshwater boundary, these transitions are relatively rare and can profoundly influence clade diversification (Vega & Wiens, 2012) and adaptation toward novel niches (i.e., niche lability; Kozak & Wiens, 2006). However, some lineages that have undergone habitat transitions seem limited by ecological constraints and exhibit patterns of niche conservatism (Betancur‐R, Ortí, Stein, Marceniuk, & Pyron, 2012; Bloom & Lovejoy, 2012; Buser, Finnegan, Summers, & Kolmann, 2019; Wiens & Graham, 2005). Understanding how transitions between marine and freshwaters influence diversification offers critical insight into the interplay between species ecology and macroevolutionary dynamics (McPeek, 2007; Weber, Wagner, Best, Harmon, & Matthews, 2017). Transitions between major aquatic habitats can alter the adaptive landscape and catalyze lineage and morphological diversification (Bloom, Weir, Piller, & Lovejoy, 2013; Guinot & Cavin, 2015; Price et al., 2011).

The habitat occupied by a species plays a key role in determining its adaptive landscape (Mahler, Ingram, Revell, & Losos, 2013). Moving to new habitats can expose taxa to new adaptive optima, in turn leading to diversification and the evolution of ecological novelty (Martin & Wainwright, 2013). For example, fishes inhabiting structurally complex coral reefs exhibit increased morphological diversity and elevated rates of evolution (Price et al., 2011, 2013). In Neotropical cichlids, both feeding morphology and body shape diversification followed transitions into new habitats (Arbour & López‐Fernández, 2013, 2014), while the fundamental locomotor bauplan of these fishes changed as well, sometimes decoupled from that of feeding morphology (Astudillo‐Clavijo, Arbour, & López‐Fernández, 2015). In shallow reefs and rivers, fishes often exhibit recurrent diversification along a bentho‐pelagic axis (Burress, Holcomb, Tan, & Armbruster, 2017; Hulsey et al., 2013; Rutschmann et al., 2012), where habitat complexity and accompanying ecological diversity drive feeding, locomotor, and body shape diversification (Hodge et al., 2018; Smith, Nelson‐Maney, Parsons, Cooper, & Albertson, 2015; Tavera, Acero, & Wainwright, 2018). While several studies have investigated how transitions between marine and freshwaters influence lineage diversification (Betancur‐R, Orti, & Pyron, 2015; Bloom et al., 2013; Santini et al., 2013), few studies have compared morphological diversification between marine and freshwater lineages (Davis & Betancur‐R, 2017).

Many fish clades are restricted to either marine or freshwater habitats. However, other fish groups exhibit greater lability of habitat occupancy, with evolutionary reconstructions suggesting multiple independent transitions between marine and freshwater habitats. For example, pufferfishes (Santini et al., 2013; Yamanoue et al., 2011), drums (Lo et al., 2015), herring, longfin herrings, and anchovies (Bloom & Lovejoy, 2012; Bloom & Lovejoy, 2014), sculpins and other cottoid fishes (Buser et al., 2019), stingrays, and needlefishes (Bloom & Lovejoy, 2017) include both marine species and freshwater species distributed across multiple continents. These trans‐marine/freshwater clades provide optimal study systems for understanding how habitat shifts alter the adaptive landscape and drive the evolution of ecological novelty and morphological disparity (Davis, Unmack, Pusey, Pearson, & Morgan, 2014).

Needlefishes (Belonidae) are typically elongate piscivorous mesopredators that swim just below the water's surface. They are distributed globally in subtropical and tropical marine, brackish, and freshwater environments, and fossil evidence suggests these fishes have been persistent predators in these waters for 8–10 million years (de Sant'Anna, Collette, & Godfrey, 2013). Several species occur exclusively in freshwater rivers of South America, Central America, and Southeast Asia. They exhibit considerable body size variation, ranging in length from the 5.0 cm freshwater *Belonion apodion* (Collette, 1966) to pelagic marine species that reach up to 2.0 m, such as *Tylosurus crocodilus* (Péron & Lesueur, 1821) and *Ablennes hians* (Valenciennes, 1846) (Collette, 2003). The repeated invasions of freshwater by marine beloniformes on multiple continents, their variation in body size and shape, and putative ecological novelty in riverine habitats (Collette, 1966; Goulding & Carvalho, 1983; Lovejoy & De Araújo, 2000) make them an excellent study system for examining morphological diversification associated with habitat transitions.

Here, we investigated how habitat transitions have affected morphological diversification in needlefishes. We analyzed body shape and size, including functional features such as fin placement, body tapering, and skull shape, and used micro‐computed tomography scanning to assess axial skeleton morphology. Our objectives were fourfold: (a) to describe the primary axes of body shape and size variation in needlefishes, (b) to test for differences in morphological diversity between marine and freshwater taxa, (c) to test for differences in rates and patterns of morphological evolution between marine and freshwater taxa, and (d) to determine whether evolutionary transitions between marine and freshwaters alter macroevolutionary adaptive landscapes. Our study demonstrates that needlefishes have experienced divergent selective regimes as a result of habitat transitions.

## 2 METHODS

### 2.1 Taxon sampling

**Figure 1:**
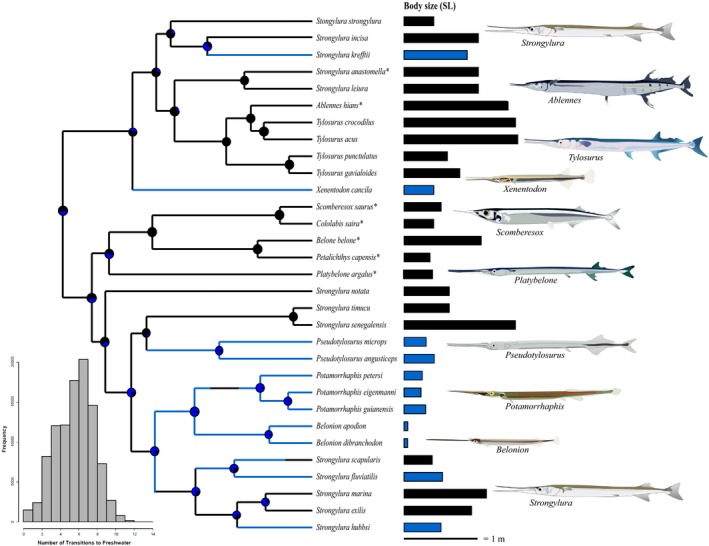
Trimmed phylogenetic tree from Bloom and Lovejoy (2017) used in analyses. Black taxa are marine species, while blue taxa are freshwater. Bar plot on right side shows maximum size for each species. Taxa with * indicate pelagic, offshore species. Body size is given as maximum standard length (cm) recorded for each taxon for ease of viewing (analyses were conducted on log‐transformed standard length). Branch lengths are proportional to time. Histogram represents the number of transitions into freshwater inferred from our SIMMAP reconstructions across the entire posterior distribution of the phylogeny

We acquired 97 specimens representing 31 of the 37 (84%) species in Belonidae, including twenty marine and eleven freshwater taxa (Table 1; Froese & Pauly, 2014; Bloom & Lovejoy, 2017). Phylogenetic analyses (Aschliman, Tibbetts, & Collette, 2005; Bloom & Lovejoy, 2017; Lovejoy, 2000; Lovejoy, Iranpour, & Collette, 2004) indicate that the sauries (previously classified in the separate family Scomberesocidae) are nested within the needlefishes (Figure 1), and hereafter, we treat the sauries as members of the needlefish (Belonidae) clade. Specimens were obtained on loan from museum collections at the Academy of Natural Sciences of Drexel University (ANSP), Auburn University Museum (AUM), the California Academy of Science (CAS), the Cornell University Museum of Vertebrates (CUMV), Smithsonian National Museum of Natural History (USNM), and University of Washington's Burke Museum (UW).

**Table 1:** List of museum specimens used in this study, their habitat affiliation, and locality data

Family	Species	Museum	ID#	SW/FW	Locality
Belonidae	*Ablennes hians*	ANSP	112005	SW	Bahamas
Belonidae	*Ablennes hians*	ANSP	112006	SW	Bahamas
Belonidae	*Belone belone*	CAS‐SU	2676	SW	Italy, Veneto
Belonidae	*Belone svetovidovi*	CUMV	CU78066	SW	Ireland
Belonidae	*Belone svetovidovi*	CUMV	CU78067	SW	Ireland
Belonidae	*Belonion apodion*	USNM	NM216734	FW	S America
Belonidae	*Belonion apodion*	USNM	NM216734	FW	S America
Belonidae	*Belonion apodion*	USNM	NM216734	FW	S America
Belonidae	*Belonion dibranchodon*	CUMV	78499	FW	Amazonas, VZ
Belonidae	*Belonion dibranchodon*	CUMV	78499	FW	Amazonas, VZ
Scomberesocidae	*Cololabis adocetus*	CAS‐SU	228232	SW	NW & C.Pacific
Scomberesocidae	*Cololabis adocetus*	CAS‐SU	228232	SW	NW & C.Pacific
Scomberesocidae	*Cololabis adocetus*	CAS‐SU	228232	SW	NW & C.Pacific
Scomberesocidae	*Cololabis saira*	ANSP	88978	SW	Mexico
Scomberesocidae	*Cololabis saira*	ANSP	88979	SW	Mexico
Scomberesocidae	*Cololabis saira*	CAS‐SU	47457	SW	No data
Scomberesocidae	*Cololabis saira*	CAS‐SU	47457	SW	No data
Scomberesocidae	*Cololabis saira*	UW	NA	SW	No data
Scomberesocidae	*Cololabis saira*	UW	NA	SW	No data
Scomberesocidae	*Cololabis saira*	UW	NA	SW	No data
Belonidae	*Petalichthys capensis*	USNM	227650	SW	South Africa
Belonidae	*Platybelone argalus*	USNM	405799	SW	Turks and Caicos
Belonidae	*Potamorrhaphis eigenmanni*	CUMV	77951	FW	Beni, Bolivia
Belonidae	*Potamorrhaphis eigenmanni*	CUMV	77952	FW	Beni, Bolivia
Belonidae	*Potamorrhaphis eigenmanni*	CUMV	77952	FW	Beni, Bolivia
Belonidae	*Potamorrhaphis guianensis*	CUMV	76874	FW	Apure, VZ
Belonidae	*Potamorrhaphis guianensis*	CUMV	76874	FW	Apure, VZ
Belonidae	*Potamorrhaphis guianensis*	CAS‐SU	14376	FW	Bolivia, El Beni
Belonidae	*Potamorrhaphis petersi*	CUMV	78500	FW	Amazonas, VZ
Belonidae	*Pseudotylosurus angusticeps*	CUMV	78505	FW	Napo, Ecuador
Belonidae	*Pseudotylosurus angusticeps*	CUMV	78505	FW	Napo, Ecuador
Belonidae	*Pseudotylosurus microps*	USNM	308327	FW	Brazil
Scomberesocidae	*Scomberesox (forsteri) saurus*	CAS‐SU	23044	SW	Chile
Scomberesocidae	*Scomberesox (forsteri) saurus*	CAS‐SU	23044	SW	Chile
Scomberesocidae	*Scomberesox (forsteri) saurus*	CAS‐SU	23044	SW	Chile
Scomberesocidae	*Scomberesox (forsteri) saurus*	CAS‐SU	23044	SW	Chile
Scomberesocidae	*Scomberesox saurus*	ANSP	7549	SW	USA
Scomberesocidae	*Scomberesox saurus*	ANSP	7549	SW	USA
Belonidae	*Strongylura anastomella*	ANSP	31698	SW	Japan
Belonidae	*Strongylura anastomella*	ANSP	31698	SW	Japan
Belonidae	*Strongylura anastomella*	CAS‐SU	80731	SW	Japan
Belonidae	*Strongylura exilis*	ANSP	81157	SW	Galapagos
Belonidae	*Strongylura exilis*	ANSP	81158	SW	Galapagos
Belonidae	*Strongylura exilis*	CAS‐SU	80722	SW	Mexico, Baja California
Belonidae	*Strongylura fluviatilis*	CUMV	78507	FW	Esmeraldas, Ecuador
Belonidae	*Strongylura fluviatilis*	CUMV	78507	FW	Esmeraldas, Ecuador
Belonidae	*Strongylura fluviatilis*	CAS‐SU	11605	FW	Colombia, Choco
Belonidae	*Strongylura hubbsi*	CUMV	77876	FW	Peten, Guatemala
Belonidae	*Strongylura hubbsi*	CUMV	77876	FW	Peten, Guatemala
Belonidae	*Strongylura hubbsi*	CUMV	77876	FW	Peten, Guatemala
Belonidae	*Strongylura incisa*	CUMV	77842	SW	Bunaken, Indonesia
Belonidae	*Strongylura incisa*	CUMV	77842	SW	Bunaken, Indonesia
Belonidae	*Strongylura incisa*	CAS‐SU	80712	SW	Micronesia, Pohnpei
Belonidae	*Strongylura incisa*	CAS‐SU	80712	SW	Micronesia, Pohnpei
Belonidae	*Strongylura krefftii*	USNM	402048	FW	Australia
Belonidae	*Strongylura leiura*	ANSP	87288	SW	Thailand
Belonidae	*Strongylura leiura*	ANSP	87289	SW	Thailand
Belonidae	*Strongylura leiura*	CAS‐SU	80698	SW	Thailand
Belonidae	*Strongylura marina*	USNM	125913	SW	USA
Belonidae	*Strongylura marina*	CAS‐SU	27598	SW	USA, Massachusetts
Belonidae	*Strongylura marina*	CAS‐SU	27598	SW	USA, Massachusetts
Belonidae	*Strongylura marina*	CAS‐SU	27598	SW	USA, Massachusetts
Belonidae	*Strongylura marina*	CAS‐SU	27598	SW	USA, Massachusetts
Belonidae	*Strongylura marina*	CAS‐SU	27598	SW	USA, Massachusetts
Belonidae	*Strongylura notata*	CUMV	CU75110	SW	Florida
Belonidae	*Strongylura notata*	CAS‐SU	35515	SW	USA, Florida
Belonidae	*Strongylura notata*	CAS‐SU	35515	SW	USA, Florida
Belonidae	*Strongylura scapularis*	USNM	206674	SW	Colombia
Belonidae	*Strongylura scapularis*	CAS‐SU	6963	SW	C. America, Panama
Belonidae	*Strongylura senegalensis*	USNM	348315	SW	Ghana
Belonidae	*Strongylura senegalensis*	CAS‐SU	80693	SW	Atlantic, Ghana
Belonidae	*Strongylura senegalensis*	CAS‐SU	80693	SW	Atlantic, Ghana
Belonidae	*Strongylura strongylura*	CUMV	78065	SW	Bolinao, Philippines
Belonidae	*Strongylura strongylura*	CAS‐SU	80690	SW	Thailand, Ranong
Belonidae	*Strongylura strongylura*	CAS‐SU	80688	SW	Thailand, Trat
Belonidae	*Strongylura strongylura*	CAS‐SU	80690	SW	Thailand, Ranong
Belonidae	*Strongylura timucu*	CUMV	CU75113	SW	Florida
Belonidae	*Strongylura timucu*	CUMV	CU75113	SW	Florida
Belonidae	*Strongylura timucu*	CAS‐SU	18570	SW	C.America, Panama
Belonidae	*Strongylura urvillii*	CAS‐SU	21701	SW	Philippines
Belonidae	*Strongylura urvillii*	CAS‐SU	21702	SW	Philippines
Belonidae	*Strongylura urvillii*	CAS‐SU	21701	SW	Philippines
Belonidae	*Tylosurus acus*	CUMV	75116	SW	Florida
Belonidae	*Tylosurus acus*	CUMV	75116	SW	Florida
Belonidae	*Tylosurus acus*	CUMV	75116	SW	Florida
Belonidae	*Tylosurus crocodilus*	USNM	347836	SW	Philippines
Belonidae	*Tylosurus crocodilus (cf)*	ANSP	90340	SW	Bahamas
Belonidae	*Tylosurus gavialoides*	USNM	211805	SW	Australia
Belonidae	*Tylosurus punctulatus*	CAS‐SU	28491	SW	Philippines, Sulu
Belonidae	*Tylosurus punctulatus*	CAS‐SU	28491	SW	Philippines, Sulu
Belonidae	*Tylosurus punctulatus*	CAS‐SU	28491	SW	Philippines, Sulu
Belonidae	*Tylosurus punctulatus*	CAS‐SU	28491	SW	Philippines, Sulu
Belonidae	*Tylosurus punctulatus*	CAS‐SU	28491	SW	Philippines, Sulu
Belonidae	*Tylosurus punctulatus*	CAS‐SU	28491	SW	Philippines, Sulu
Belonidae	*Xenentodon cancila*	CUMV	77144	FW	Bengal, India
Belonidae	*Xenentodon cancila*	CUMV	77145	FW	Bengal, India
Belonidae	*Xenentodon cancila*	CUMV	77145	FW	Bengal, India

### 2.2 Image acquisition and geometric morphometric analyses of body shape

**Figure 2:**
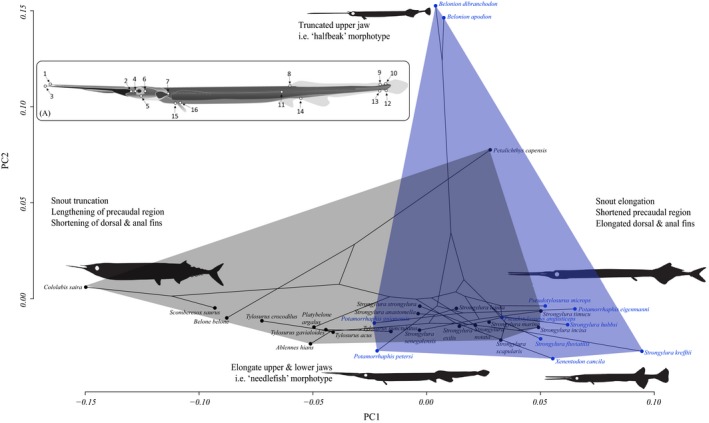
Phylomorphospace for needlefishes generated from principal component axes (PC 1, 2) and landmarked diagram of *Potamorrhaphis* (bottom). Convex hulls indicate morphospaces of freshwater (blue) and marine (gray) taxa. Text describes changes in body shape at respective extremes of the PC axes. Inset (A) gives the landmark positions used with these specimens. Landmark positions: 1—anterior extent of maxilla, 2—jaw joint, 3—anterior extent of dentary, 4—anterior extent of orbit, 5—ventral extent of orbit, 6—posterior extent of orbit, 7—posterior extent of operculum, 8—anterior insertion of first soft dorsal fin ray, 9—posterior insertion of dorsal fin, 10—dorsal insertion of caudal fin, 11—posterior limit of the caudal peduncle, 12—ventral insertion of caudal fin, 13—posterior insertion of anal fin, 14—anterior insertion of anal spine, 15—ventral insertion of pectoral fin, 16—dorsal insertion of pectoral fin

We photographed whole specimens against a foam background using insect pins or held beneath a glass panel to minimize artifacts from warping and twisting. Images of whole needlefishes were landmarked (Figure 2) using the program tpsDig2 (v. 2.31; Rohlf, 2004). All further analyses were performed using R (v. 3.4.4; https://www.r-project.org/). We carried out a Generalized Procrustes Analysis (GPA) on specimen landmark data using the *gpagen* function (geomorph package, v. 3.0.5; Adams, Collyer, Kaliontzopoulou, & Sherratt, 2016). GPA standardizes landmark configurations among specimens with respect to rotation, scale, and translation, explicitly quantifying body shape and body size, independently. The GPA dataset was then ordinated using principal components analysis (PCA) (*plotTangentSpace*; Adams et al., 2016), which reduces dataset dimensionality and generates a trait morphospace. We also tested for a significant effect of size on shape using Procrustes regression with permutation (×1,000) (*procD.allometry*; Adams & Collyer, 2018), which estimates the effect of centroid size on our Procrustes‐aligned shape coordinates. To test whether freshwater needlefish have fundamentally different body shapes than saltwater needlefish, we used a Procrustes ANOVA in a phylogenetic framework, which uses permutation procedures to assess statistical hypotheses under a Brownian motion model of evolution (*procD.pgls*; Adams & Collyer, 2018).

### 2.3 Computed tomography scanning and axial skeleton meristics

Macroevolutionary changes in body size in fishes frequently involve alterations to the number, spacing, or size of vertebral elements, particularly in slender elongate fishes (Ward & Mehta, 2010). We used µCT scanning to examine gross morphological changes occurring in the axial skeleton of beloniformes in relation to habitat transitions. Each specimen was labeled and photographed with a scale bar prior to scanning. Specimens were then wrapped in ethanol‐soaked cheesecloth to prevent desiccation, packed together within a PVA‐plastic cylinder, and covered in plastic wrap. Most specimens were scanned using the 1173 Bruker SkyScan µCT system at the Karel Liem Bioimaging Center at Friday Harbor Laboratories, while several larger specimens were scanned using the University of Washington Engineering Department's NSI V‐Tek 5000 µCT scanner (Seattle, WA). All smaller specimens were scanned at 65 kV, 123 µA, and a 1160‐ms exposure, using a 1‐mm aluminum filter and a 0.3°‐0.4° rotation step. Resolution varied from 15 to 35 µm, and raw image stacks in.bmp format were reconstructed using NRecon (Bruker Corp.) as.jpeg images. Images were then converted to DICOM file format for viewing in CTVox 2.7 software (Bruker Corp.) and segmented using the program Horos (The Horos Project, 2015 http://www.horosproject.org/). We counted the number of vertebrae for each CT‐scanned specimen and then averaged vertebral counts between individuals from the same species. We obtained maximum recorded standard lengths for each species from FishBase (Froese & Pauly, 2014) and log_10_‐transformed them for further analyses. We then performed phylogenetically explicit ANOVA (Revell, 2009) using the function *phylANOVA* (phytools v. 0.6‐99 package; Revell, 2012), which uses phylogenetic simulations to test whether (logged) maximum recorded lengths and mean vertebral counts differed statistically (*α* < .05) between marine and freshwater taxa. The phylogeny used for analyses is discussed below.

### 2.4 Phylogeny

For all comparative analyses, we used the time‐calibrated phylogeny for Beloniformes from Bloom and Lovejoy (2017), which is based on a multigene dataset (*cytb*, *rag*1, *rag*2, *tmo‐4c4*) of 3,318 base pairs for 104 species and represents the most densely sampled phylogeny for this group. We used the *drop.tip* function to trim the phylogeny to include only the species in our morphological dataset (*n* = 31). We then inferred the evolutionary history of habitat using stochastic character mapping (Bollback, 2006; Huelsenbeck, Nielsen, & Bollback, 2003) with the *make.simmap* function (phytools; Revell, 2012; Figure 1) for 1,000 trees. The evolutionary history of habitat was reconstructed on 1,000 random trees using the posterior distribution from Bloom and Lovejoy (2017) to account for phylogenetic uncertainty. To assess the best model for the transition matrix, we fitted the following models: (a) a model allowing for equivalent rates of transition for both freshwater and marine lineages (“ER”) and (b) a model allowing these rates to vary (“ARD” or “all rates different”) using the function *ace* in the package ape (v. 5.3; Paradis, Claude, & Strimmer, 2004). We then compared the two models (ER vs. ARD) using a likelihood‐ratio test and found that the ER was the best‐supported model. We used this ER model and estimated the prior distribution of the states at the root of the tree and used the MCMC option to set the parameters of the Q matrix.

### 2.5 Phylomorphospace and adaptive optima analyses

We examined whether marine and freshwater clades overlap in trait space or whether lineages are exploring alternative regions of morphospace. We used the broken stick method to determine the number of informative principal component axes to retain for analyses (*screeplot.cca* function in the package vegan). We then visualized a belonid morphospace by plotting these remaining PC axes and projected the phylogeny onto species values to form a phylomorphospace (Sidlauskas, 2008), as implemented in phytools (Revell, 2012). Convex hulls were fit to marine and freshwater taxa, separately based on the method of Eddy (1977), using the *chull* function. We used *compare.evol.rates* function (from package geomorph; Adams et al., 2016) to determine whether rate shifts in the evolution of body shape are associated with habitat transitions. We iterated this process 5,000 times using phylogenetic simulation, whereby simulated tip data are obtained under Brownian motion using a common evolutionary rate pattern for all species on the phylogeny (Denton & Adams, 2015). From Adams, Collyer, Otarola‐Castillo, and Sherratt (2014) “From the data the net rate of shape evolution for each group in the multi‐dimensional space is calculated, and a ratio of rates is obtained.” Since we only compared between two groups (marine and freshwater), the ratio of the maximum to minimum rate was not used as a test statistic.

We tested three evolutionary models in the package OUwie (v. 1.5; Beaulieu, Jhwueng, Boettiger, & O'Meara, 2012) to determine whether freshwater and marine needlefishes evolved toward different adaptive optima with regard to body size and species' mean vertebral count. The evolutionary models were run on all 1,000 trees (from the posterior distribution) to account for uncertainty in habitat optimization. The first two evolutionary models we tested were models of Brownian motion, which assumes no trait differences between freshwater and marine lineages, with trait variation accruing randomly as a function of time. The next model, a single OU (Ornstein–Uhlenbeck) model, assumes that freshwater and marine lineages are evolving toward a shared trait optimum. The next sets of models were multi‐peak OU models, with increasing parameter complexity. The simplest multiple peak OU model was OUM, which assumes different trait optima (*θ*) for freshwater and marine lineages, but each lineage has the same pull toward the optimal trait value (*α*) and the same rate parameter (*σ* ^2^).

Model fit was evaluated using the Akaike information criterion (AIC) with a correction for small sample size (AICc; Burnham & Anderson, 2002). AICc values were calculated for each iteration and averaged across all iterations for each model. Mean AICc values were used to calculate AICc weights, and the model with the lowest AICc weight was selected as the best model. Eigen decomposition of the Hessian matrix provides an indication of whether the model search returned the maximum‐likelihood estimate (Beaulieu et al., 2012). If the eigenvalues are positive, then the results are considered reliable. To ensure that all maximum‐likelihood results were reliable, we removed any model run that returned a negative eigenvalue prior to evaluating the model fit.

OUwie uses complex OU models that cannot always be reliably detected when the statistical power is low (Boettiger, Coop, & Ralph, 2012), and low power can lead to complex OU models being incorrectly favored over models of Brownian evolution (Cooper, Thomas, & FitzJohn, 2016; Ho & Ané, 2014). To determine whether we had significant power to accurately detect the complex models, we performed 1,000 OUwie simulations for max body size and mean vertebral count using the function *OUwie.sim*. The simulated datasets were performed with the parameter estimates for the best‐fit model of each morphological character in our empirical dataset (Table 2). The simulated data were then run through all three models in OUwie to determine whether the simulated model could be accurately recovered with our sample size.

**Table 2:** Comparison of model fits and trait optima (*θ*) for body size and mean vertebra number between freshwater and marine lineages

	Model	Rank	AICc	Δ AICc	AICc weight	*θ*_fw_	*θ*_mar_	*α*_fw_	*α*_mar_	*σ* ^2^_fw_	*σ* ^2^_mar_
Body size	**OUM**	**1**	**8.8**	**0**	**0.86**	**1.16**	**1.87**	**0.052**	**0.052**	**0.007**	**0.007**
	BM1	2	13.4	4.6	0.09	—	—	—	—	0.003	0.003
	OU1	3	14.5	5.7	0.05	1.79	1.25	0.015	0.015	0.004	0.004
Average vertebral count	**OU1**	**1**	**222**	**0**	**0.356**	**68.9**	**68.9**	**0.028**	**0.028**	**4.99**	**4.99**
	BM1	2	223	0.349	0.299	—	—	—	—	2.89	2.89
	OUM	3	223	1.01	0.215	61.9	71.1	0.036	0.036	5.45	5.45

Note

Emboldened rows represent best‐fit model based on lowest AICc score. *θ*_fw_ is the estimated trait optima for freshwater species, *θ*_mar_ is the estimated trait optima for marine species, *α*_fw_ is the estimated pull toward the optimal trait value for freshwater species, *α*_mar_ is the estimated pull toward the optimal trait value for marine species, *σ* ^2^_fw_ is the estimated rate parameter freshwater species, and *σ* ^2^_mar_ is the estimated rate parameter for marine species.

## 3 RESULTS

Belonids have undergone transitions from marine to freshwater habitats six times, with no reversals to the marine environment (Figure 1). Correspondingly, we found biased directionality in habitat transitions; transitions from marine to freshwater habitats were almost twice as likely as freshwater to marine (7.205 vs. 4.191 changes).

**Figure 3:**
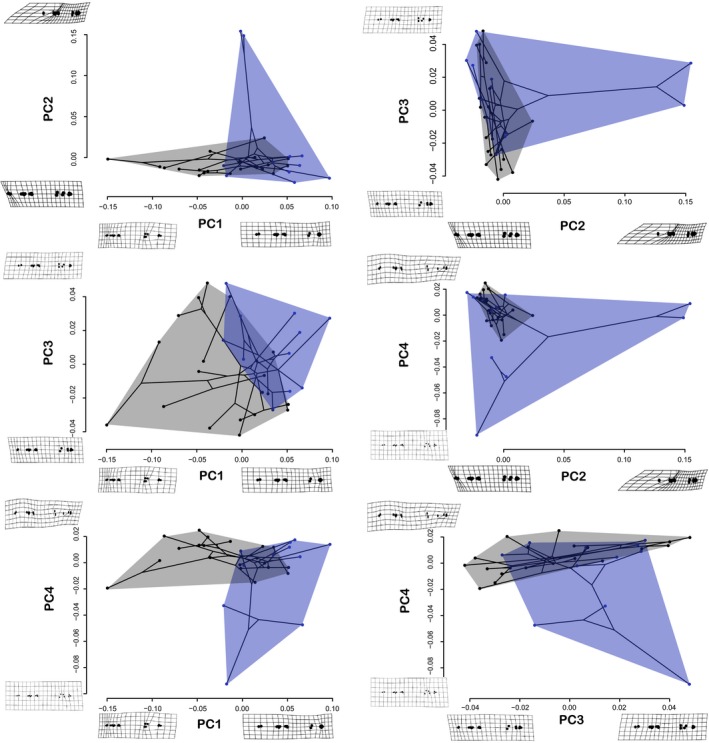
Scatterplots of paired PC axes used to generate phylomorphospaces of needlefish body shape. Warp grids are shown as examples of body shape change at axes. Convex hulls indicate morphospaces of freshwater (blue) and marine (gray) taxa

Geometric morphometric analyses of body shape found differences in body shape among the sampled marine and freshwater taxa. Observed PC eigenvalues crossed broken stick components at PC4, suggesting that PCs 1–4 have statistically significant phylogenetic signals, so we limit our discussion to these axes. The first four axes explained 45%, 26%, 11%, and 8% of total body shape variation, respectively. PC1 described relative trunk length, and dorsal fin and anal fin lengths. PC2 described the relative lengths of the upper and lower jaws (Figure 2). PC3 described the relative shape (length) of the jaws, as well as the size and placement of the dorsal and anal fins (Figure 3). PC4 predominantly described the shape of the precaudal region, specifically the distance between the caudal peduncle and the dorsal and anal fins (Figure 3). We recovered a significant effect of centroid size on body shape (*p* < .001); however, in order to retain as much biological information as possible, we chose not to correct for allometric scaling (sensu Evans, Williams, & Westneat (2019) and sources therein). Relatedly, freshwater and saltwater needlefishes had significantly different body shapes (*p* = .031; *R* ^2^ = .053) and mean centroid sizes with respect to habitat (*p* = .016; *R* ^2^ = .071), according to Procrustes ANOVA results. These results could indicate that size effects on shape or shape effects on size are driving differences between marine and freshwater taxa for either metric. Regardless, the evolutionary nature of body shape and size are inherently different between freshwater and marine taxa.

In general, marine and freshwater taxa show little variation with regard to PC2, with the notable exceptions of *Belonion* and *Petalichthys* (Regan, 1904), while species from different habitats are separated primarily along PC1 (e.g., body elongation or truncation; Claverie & Wainwright, 2014) (Figure 3). Freshwater taxa fall mostly outside the bounds of the phylomorphospace occupied by marine taxa. PC2 captured relative differences in length between the upper and lower jaws, a proxy for which species have a “halfbeak” morphotype (e.g., *Belonion*). *Belonion,* with its foreshortened upper jaw (i.e., “halfbeak” morphotype) and comparably large eyes (relative its head size), loaded positively on PC2 (Figures 2 and 3).

In most cases, convex hulls for freshwater taxa encompassed greater regions of morphospace than those of marine taxa (Figures 2 and 3). Marine and freshwater taxa occupied partially overlapping, but largely separate, regions of morphospace. Several taxa consistently appeared on the periphery of morphospace plots, namely the miniaturized Neotropical freshwater genus *Belonion* (*B. apodion* and *B. dibranchodon* Collette, 1966), the Southeast Asian freshwater needlefish *Xenentodon cancila* (Hamilton, 1822), and Neotropical freshwater *Potamorrhaphis* (*P. eigenmanni* Miranda Ribeiro, 1915 and *P. petersi* Collette, 1974). These freshwater fishes appear to be exploring novel regions of morphospace, and all are notably smaller freshwater taxa.

**Figure 4:**
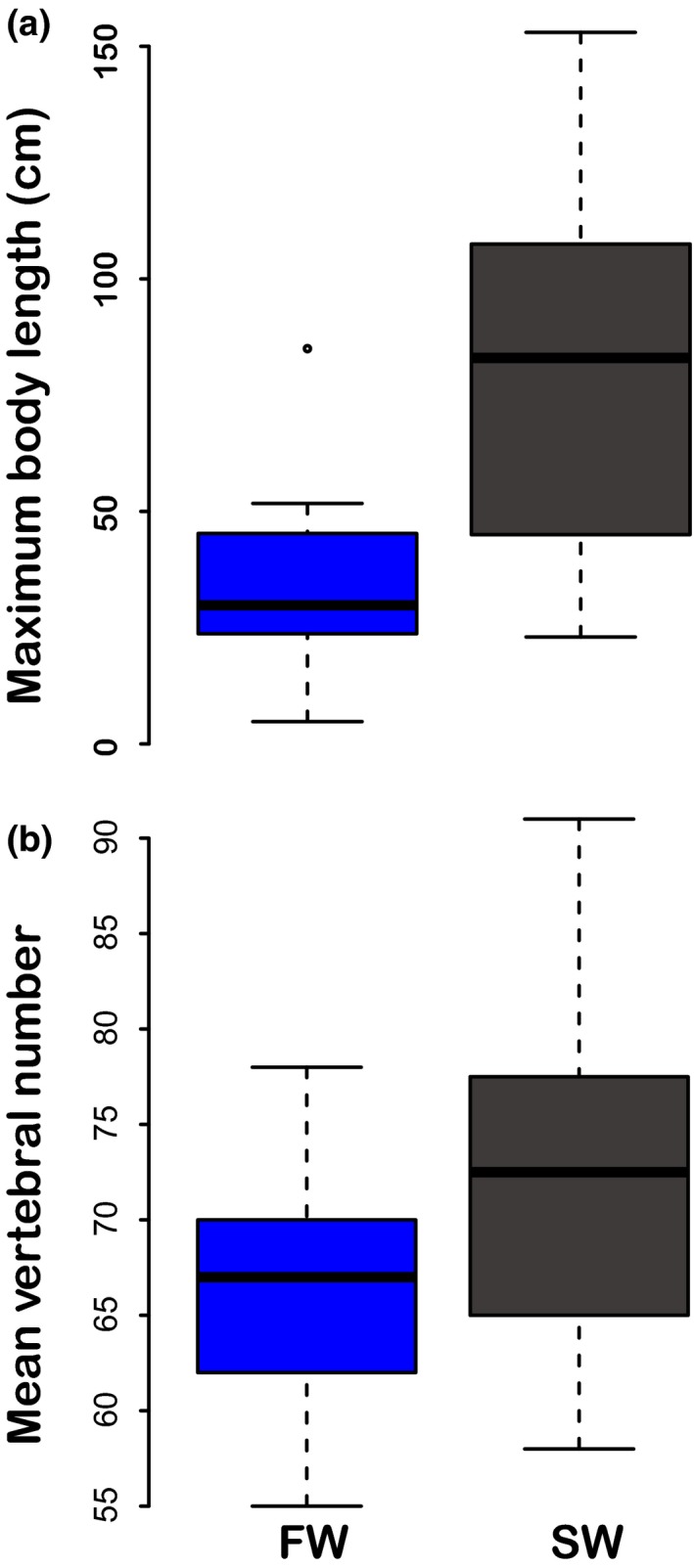
Boxplots of maximum body size (a) and average vertebral count (b) in freshwater (blue) and marine (black) beloniformes. Points represent outliers. There is a statistically significant difference in maximum body size (standard length in cm) between marine and freshwater species [*F* = 12.82, *p* = .022], but not mean vertebral counts [*F* = 4.24, *p* = .203], according to a phylogenetic ANOVA with statistical significance evaluated by phylogenetic simulation

Freshwater needlefishes were significantly smaller (reduced lengths and depths) than marine needlefishes (Figure 4). Our *phylANOVA* analyses [*α* < .05] showed a statistically significant difference in maximum body length between marine and freshwater habitats [*F* = 12.5, *df* = 29; *p* = .016]. Offshore, pelagic taxa such as *Ablennes hians* and *Tylosurus* species had larger maximum body lengths than other taxa, while freshwater taxa such as *Pseudotylosurus* Fernández‐Yépez, 1948*, Potamorrhaphis* and *Belonion* species had smaller body sizes. We did not find statistically significant differences between mean vertebral counts between species in marine versus freshwater habitats [*F* = 4.24, *df* = 29; *p* = .197]. However, some freshwater species (e.g., *Belonion*) had drastically fewer vertebrae relative to other needlefishes in general.

**Figure 5:**
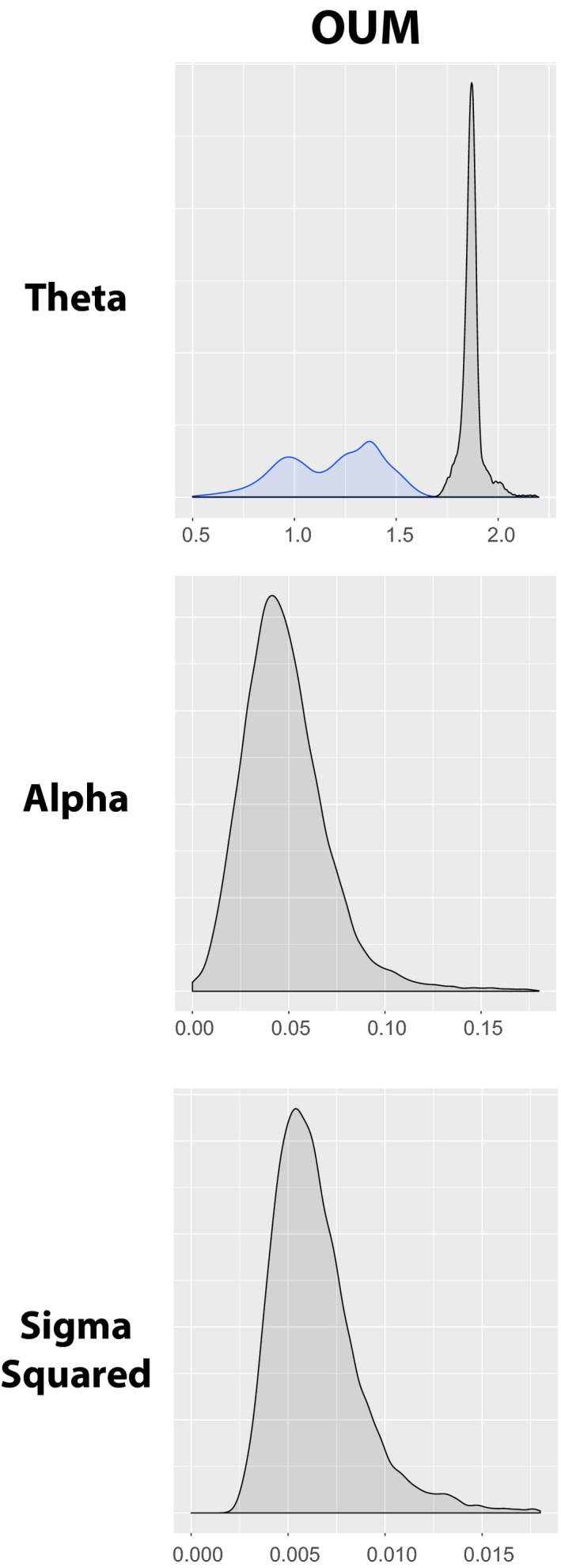
Distribution of theta, alpha, and sigma‐squared values for log maximum body size (standard length) for freshwater (blue) and marine (black) needlefish lineages for the best‐supported model (OUM) from the empirical OUwie analyses

The OUwie analyses of body size for freshwater and marine lineages indicate the best‐fit model is OUM, a model supporting different optimal trait values (*θ*) (Table 2; Figure 5). These analyses indicate that marine lineages have a larger optimal body size than freshwater lineages although there possibly are two smaller‐bodied adaptive size optima for freshwater needlefishes (Figure 5).

**Figure 6:**
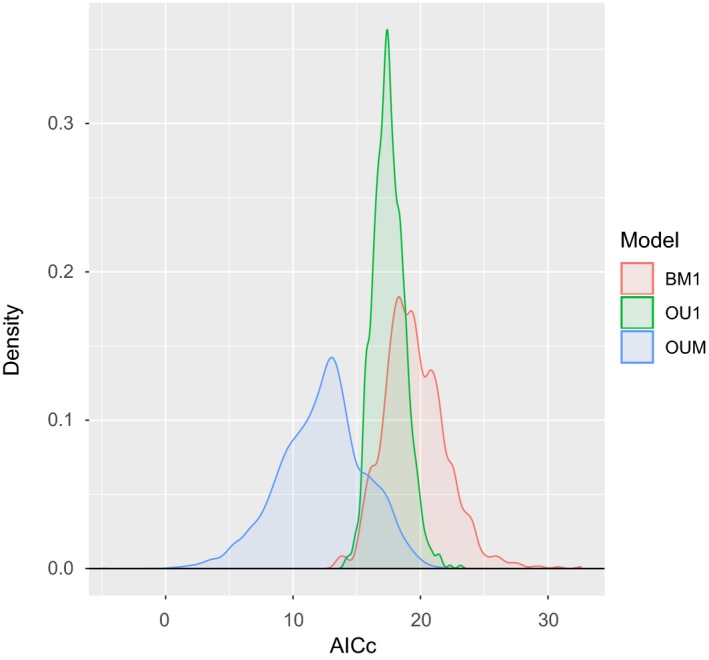
Density plot of AICc scores for the simulations for the best‐supported model (the OUM model) of log maximum body size (standard length)

Results of our simulations for body size show that our dataset has enough statistical power to clearly separate the different multi‐peak OU models from the models of Brownian motion and the single‐peak OU model (Figure 6). Furthermore, our simulations show that we could accurately recover the estimated theta in most of our simulations. Nevertheless, we can clearly discriminate between single‐peak and multi‐peak models, as well as recover the correct placement of the optimal trait values (Figure 6), allowing us to conclude that marine lineages evolved toward a larger body size than freshwater lineages (Figure 5).

The OUwie analysis on mean vertebral count supported multiple models: the OU model with a single adaptive optimum, a Brownian motion model, and a multi‐peak OU model. The failure to differentiate among these models was likely due to lack of statistical power in our dataset; therefore, we do not discuss the results of the model test for mean vertebral count.

## 4 DISCUSSION

### 4.1 Body shape and habitat

Ecological transitions among habitats clearly shape the adaptive landscape and result in both novel and repeated bauplans, outcomes that support both contingent and deterministic evolution (Blount, Lenski, & Losos, 2018). Freshwater needlefish lineages have both retained ancestral, marine bauplans and evolved radical departures from these same bauplans (e.g., *Belonion*). As a result, marine and freshwater taxa exhibit overlapping, yet staggered morphospace occupation (Figures 2 and 3). In addition, considering all phylomorphospace configurations in Figures 2 and 3, freshwater taxa are more morphologically diverse than marine taxa. This demonstrates that habitat transitions have promoted diversification of body shapes and size, as well as faster rates of shape and size evolution in belonids overall perhaps due to release of ecological limits on clade diversification in novel habitats (Betancur‐R et al., 2012; Bloom & Egan, 2018).

What are the evolutionary patterns in morphological change associated with habitat transitions? Across a myriad examples of habitat transitions, from marine Antarctic shallows, to tropical reefs and non‐reefs, or within African Rift lakes, fishes have evolved along a bentho‐pelagic axis, with deeper, laterally compressed bodies associated with complex benthic habitats and more fusiform shapes associated with open water (Hulsey et al., 2013; Rutschmann et al., 2012; Tavera et al., 2018). In contrast, we find that needlefishes in marine and freshwater exhibit niche conservatism because they have not deviated from epipelagic or limnetic habitats, typically cruising just below the water's surface (Goulding & Carvalho, 1983). Instead, we suggest that microhabitat and locomotory demands for either precise maneuvering (most freshwater taxa) or sustained swimming (many marine taxa) are key determinants of body shape evolution in needlefishes and have directed phenotypic novelty (Figures 2 and 3).

Interestingly, phenotypic novelty in freshwater needlefishes evolved independently in different geographic areas. For example, freshwater lineages including South American *Potamorrhaphis* and *Belonion*, as well as Southeast Asian *Xenentodon,* invaded novel regions of morphospace relative to marine taxa and likely in response to open niches in continental rivers (Foster, 1973; Goulding & Carvalho, 1983), as indicated by the phylomorphospace (Figure 2). Both lineages exhibit an overall shortening of the body relative to marine taxa, while also having rounded or squared caudal fins (Collette, 1966; Foster, 1973), which likely facilitate maneuvering in the highly structured habitats in which they occur, that is, smaller rivers, streams, and wetland habitats. Our OU methods detect two possible peaks in freshwater body size optima, which we propose highlights the extreme body plan novelty in *Belonion* relative to other diminutive freshwater needlefishes such as *Xenentodon* and *Potamorrhaphis* (Figure 6). *Belonion* have pectoral fins that are shifted ventrally relative to marine needlefishes and have larger eyes relative to body size (Figure 2). The small size of *Belonion* makes them poor swimmers, and they rely on crypsis for predator avoidance, hiding among floating debris such as leaves (N. R. Lovejoy, personal observation). The combination of these traits in *Belonion* suggests that to some degree, the habitat complexity of freshwater systems, for example, leaf litter, floating detritus, and overhanging branches, has allowed for ecological novelty to arise twice in freshwater needlefishes.

Conversely, other freshwater needlefishes such as the South American genus *Pseudotylosurus* occupy open‐water habitats in medium to large river systems, maintain a highly piscivorous trophic niche, and retain a bauplan consistent with marine species. Thus, they largely continue functioning like coastal or open‐ocean needlefishes (Lovejoy & Collette, 2001), suggesting a degree of ecological conservatism despite undergoing a major transition between marine and freshwaters. *Pseudotylosurus* species are large fish with an elongate caudal region and forked tails (Figures 2 and 3), typical of species that continuously cruise open waters, attacking prey with quick lunges (Webb, 1984). Taxa superficially like *Pseudotylosurus*, including *Tylosurus*, *Petalichthys*, and *Ablennes*, occur in similar regions of the phylomorphospace, reside in reef and pelagic marine habitats, and tend to consume considerably larger prey than most coastal or freshwater belonids; correspondingly, these taxa generally have shorter, robust jaws.

### 4.2 Body size evolution

**Figure 7:**
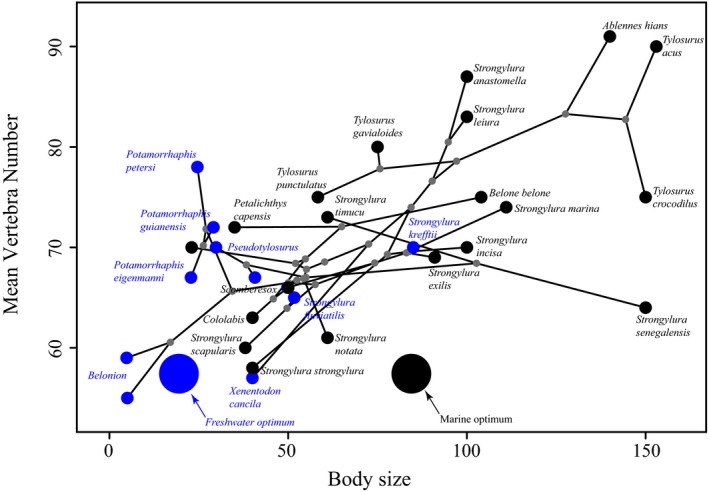
Scatterplot of two variables, body size (standard length in cm) and vertebral counts, plotted with a phylogeny projected onto it using ancestral state construction for node positions (i.e., phylomorphospace). Marine (black) lineages evolve larger body sizes but have similar vertebral counts compared with most freshwater (blue) lineages. Large black and blue circles represent the location of clade trait optima for each habitat type as determined by the best‐fitting evolutionary model. Body size optima (particularly for marine taxa) are small likely because (1) high degree of scatter in body sizes for marine taxa and (2) the ancestral theta was likely smaller, due to the combined input of both marine (larger) and freshwater (smaller) taxa pulling the respective values of thetas to be smaller

Freshwater belonids have evolved smaller body sizes than marine belonids (Figures 4 and 7), demonstrating that an organism's ecology can have profound effects on phenotypic macroevolution (Bloom, Burns, & Schriever, 2018; Collar, Schulte Ii, & Losos, 2011). Smaller body sizes in freshwater taxa have been widely reported, with explanations ranging from smaller sizes offering greater maneuverability in structured environments (Ward & Azizi, 2004; Webb, 1982) or simply reducing energetic demands in size‐constrained or complex microhabitats (Weitzman & Vari, 1988). Many pelagic marine fishes likely maintain larger size to migrate large distances, evade, or outgrow open‐ocean predators (e.g., tunas, billfishes, and sharks), and pursue elusive, strong‐swimming prey (Webb, 1984). Smaller body sizes and left‐skewed body size distributions of freshwater fish communities (Griffiths, 2012) appear to be a feature of both Neotropical primary freshwater fishes (Steele & López‐Fernández, 2014) and invaders of freshwater such as needlefishes, anchovies (Bloom, Kolmann, Foster, & Watrous, 2020; Roberts, 1984), pufferfishes (Santini et al., 2013), and stingrays (Carvalho, Rosa, & Araújo, 2016; Monkolprasit & Roberts, 1990). Our study provides additional evidence that selection toward size‐related adaptive peaks can be strong (Bloom et al., 2018; Burns & Bloom, 2020), possibly because body size covaries with many other phenotypic and life history traits (Romanuk, Hayward, & Hutchings, 2011), offering multiple selective surfaces (Peters, 1986).

### 4.3 Vertebrae evolution

In contrast to our body size data, we did not detect differences in mean vertebral count (Figure 4) or adaptive optima (Figure 7) between marine and freshwater belonids. This is surprising given the documented correlation between vertebral counts and body size in fishes (Lindsey, 1975; Ward & Brainerd, 2007). Ward and Brainerd (2007) surveyed seven actinopterygian clades and showed that variability in cranial elongation was negligible compared to variability in the axial skeleton for explaining body length. However, Ward and Mehta (2010) reported that body length is often positively correlated with head length. In the case of belonids, we found that changes in absolute body length in belonids can stem from either elongation or truncation of their needle‐like jaws, or similar changes to the axial skeleton. The evolutionary and developmental plasticity of skull morphology observed in beloniformes might make the crania more amenable to modification, while changes to the number of axial skeletal elements (centra) appear more static.

An interesting case of potential contrasting mechanisms for the evolution of vertebrae was observed in the Neotropical freshwater lineage composed of *Potamorrhaphis* and *Belonion*. Both genera are smaller than average marine taxa (including their marine sister lineage). However, *Potamorrhaphis* has vertebral counts that are typical of marine belonids (66–78), while miniaturized *Belonion* have drastically fewer vertebrae (55–59). These data suggest that two distinct mechanisms for body size reduction may be acting on freshwater needlefish bauplans: (a) proportional dwarfism, whereby taxa such as *Potamorrhaphis* are “miniaturized” versions of marine relatives, or (b) mosaic heterochrony in the case of *Belonion* (Alberch, Gould, Oster, & Wake, 1979). In the latter scenario, *Belonion* may have lost vertebral elements as a result of developmental truncation. Supporting this idea is the fact that *Belonion* matures at miniscule sizes (5 cm), has an elongated lower jaw but short upper jaw (observed in many subadult needlefish species; Lovejoy et al., 2004), and has lost or reduced both axial and appendicular skeletal elements (Collette, 1966). Overall, these findings suggest strong selection for reduced body sizes in Neotropical freshwater taxa (Weitzman & Vari, 1988) and multiple means by which that selection can effect changes (Bloom et al., 2020).

## CONFLICT OF INTEREST

The authors declare no conflicts of interest.

## AUTHOR CONTRIBUTIONS

Matthew A. Kolmann, Devin D. Bloom, and Justin Ng conceived and designed the study. Justin Ng and Matthew A. Kolmann collected the data. Matthew A. Kolmann, Justin Ng, and Michael D. Burns analyzed the data. Matthew A. Kolmann, Nathan R. Lovejoy, Justin Ng, Michael D. Burns, and Devin D. Bloom drafted the initial version of the manuscript. All authors contributed to later versions of the manuscript. **Matthew A. Kolmann:** Conceptualization (equal); formal analysis (equal); funding acquisition (equal); investigation (equal); methodology (equal); project administration (lead); visualization (equal); writing – original draft (lead); writing – review and editing (equal). **Michael D. Burns:** Conceptualization (equal); formal analysis (equal); funding acquisition (equal); investigation (equal); methodology (equal); project administration (equal); visualization (equal); writing – review and editing (equal). **Justin Ng:** Conceptualization (equal); formal analysis (equal); funding acquisition (equal); investigation (equal); methodology (equal); project administration (equal); visualization (equal); writing – original draft (equal); writing – review and editing (equal). **Devin D. Bloom:** Conceptualization (equal); data curation (equal); formal analysis (equal); funding acquisition (equal); investigation (equal); methodology (equal); project administration (equal); writing – original draft (equal); writing – review and editing (equal). **Nathan R. Lovejoy:** Conceptualization (equal); data curation (equal); formal analysis (equal); funding acquisition (equal); investigation (equal); methodology (equal); project administration (equal); writing – review and editing (equal).

## Data Availability

Sequence data are deposited in GenBank and supplemented by data from Bloom and Lovejoy (2017) https://doi.org/10.1111/jbi.12954. Other phylogeny and tree files are available after request from the authors (DDB). Matrix of maximum body sizes (standard length in cm), vertebral counts, and habitat affiliation are available online at Dryad https://doi.org/10.5061/dryad.66t1g1jzc.
